# Literary Medicos

**Published:** 1920-01-31

**Authors:** 


### LITERARY MEDICOS.

### Doctors Famous in Other Spheres.

A lecture of much interest and charm was given to the members of the Leeds Medical Society last week by Sir Berkeley Moynihan on those who have practised in or belonged to the medical profession and have forsaken it and achieved distinction in other walks of life. He took first of all politics, mentioning Dr. Addison, Minister of Health, noted as a student for the frequency of his absence from lectures, and for an important paper written many years ago on the topographical anatomy of the abdominal viscera. Dr. Jameson was another instance, and the greatest of all was declared to be M. Clemenceau, the ex-Premier of France, who entered the profession by attaining the minimum number of marks necessary. Incidentally, M. Clemenceau has also written a novel now being reviewed in the English papers.

In literature there are a number of distinguished medical "truants," as Sir Berkeley describes them. One of the greatest masters of English prose, Sir Thomas Clifford Allbutt, was once on the staff of the Leeds General Infirmary. Goethe, Goldsmith, Schiller, George Crabbe, Smollett, Mr. Somerset Maugham, Professor Henry Morley, and the Poet Laureate (Dr. Bridges) all turned from medicine to literature with success. That delightful American essayist, Doctor Oliver Wendell Holmes, also wrote one of the greatest essays ever written in the history of medicine—"Puerperal fever a private pestilence." For the first time he pointed out that puerperal fever was a contagious disease. Among modern novelists are Conan Doyle and Warwick Deeping.

Other famous "truants" were Sir Joseph Hooker, and Huxley in science; Livingstone, Mungo Park, and "Chinese" Morrison in exploration; and W. G. Grace, Poulton Palmer (whose father has just written a notable memoir of this fine sportsman's individuality), Leonard Stoker, and Joshua Pim.

Sir Berkeley Moynihan was facetious at the expense of some novelists in their handling of medical matters and disease. Experts must be the bane of the imaginative writer. Ask a naturalist for his views on Goldsmith's "Animated Nature." His list could easily be further extended: Charles Wyndham, Keats, Professor Tonks, Sir Seymour Haden, Sir John Kirk (of Zanzibar), Sir Thomas Browne, Lord (Chancellor) Finlay, and the author of "Rab, and his Friends," are additions to it which certainly should not be overlooked.

